# Family context, directed and random exploration profiles, and child socioemotional functioning

**DOI:** 10.1017/S0954579426101734

**Published:** 2026-07-17

**Authors:** Zhi Li, Yuanzhe Li, Patrick T. Davies, Melissa L. Sturge-Apple

**Affiliations:** 1 Mt.Hope Family Center, University of Rochester, USA; 2 Amazon.com Inc, USA; 3 Department of Psychology, University of Rochester, USA; 4 Warner School of Education, University of Rochester, USA

**Keywords:** computational modeling, exploration–exploitation, family context, socioemotional functioning, evolutionary developmental perspective, environmental adversity

## Abstract

Guided by the evolutionary developmental perspective, this multi-method, longitudinal study seeks to examine “hyperparameters” that organize young children’s learning. In particular, we adopt computational modeling and a person-centered approach to characterize children’s exploration–exploitation profiles during a reward probability task. We also explored the family socioeconomic precursors and child psychological sequelae of the profiles. Participants were 243 families with a preschooler (Mean age = 4.60 years, 56% girls) from diverse racial and ethnic backgrounds (46% Black, 19% Latinx). Family socioeconomic risks were measured at wave one, and exploration–exploitation and socioemotional functioning were assessed two years later via a reward probability task and teacher report, respectively. Person-centered approach revealed three exploration–exploitation profiles, reflecting (a) a high-random exploration, (b) a high-directed exploration, and (c) a balanced exploration–exploitation profile. Greater family socioeconomic risk was linked to an elevated likelihood of membership in the high-random exploration profile (e.g., Z = −2.37). In turn, the high-random exploration profile exhibited the highest attention problems and greater externalizing problems. Findings have critical implications for understanding the processes through which children adapt to adverse family contexts.

Life history theory stipulates that individuals allocate their limited resources toward specific survival goals at the relative expense of others (e.g., growth vs. reproduction; Belsky et al., 1991; Del Giudice et al., 2015). These trade-off decisions shape the rate of development along a slow versus fast continuum and are presumed to make strategic sense in adapting to the anticipated environment and maximizing one’s reproductive fitness. Exposure to childhood adversity may accelerate life-history strategy, indicated by greater risk-taking, impulsivity, opportunistic advantage-taking interpersonal orientations that reflect poor social competence (Doom et al., 2016; Li et al., 2018), and problematic behavior (e.g., Doom et al., 2016) in literature traditionally informed by evolutionary life-history strategy.

Notably, Frankenhuis and Gopnik (2023) research syntheses called for bridging different areas (i.e., cognitive science, evolutionary developmental perspective) by including “*hyperparameters*” *as additions to the traditional life-history traits*. Hyperparameters consist of higher-order processes that organize learning based on earlier experiences. This conceptualization is also supported by the meta-learning framework (Nussenbaum & Hartley, 2024), which highlights that developmental experiences (e.g., early adversity) may shape the “outer-loop” of the learning process (i.e., learning operating through long developmental timescales) and slowly adjust the person’s meta-parameter [similar to the “hyperparameters” concept advanced by Frankenhuis and Gopnik (2023)] to adapt to the experienced environment. These meta-parameters of learning, in turn, govern the “inner-loop” learning and decision-making process, manifested by the task-specific learning patterns in the trial-by-trial behavior. Crucially, while these “outer-loop” hyperparameters are difficult to measure directly, the task-specific “inner-loop” parameters derived from computational modeling may serve as measurable proxies. We conceptualize the parameters estimated in our study as the task-specific manifestations of these broader, developmentally shaped hyperparameters.

## Exploration versus exploitation profiles

To understand how these hyperparameters function, it is necessary to define the fundamental trade-offs that organisms face between exploration and exploitation. Exploration involves searching widely through a space of possibilities – gathering information about the environment, even if it results in errors and temporary reductions in performance. In contrast, exploitation involves narrowing the search to capitalize on known rewards, prioritizing efficiency and immediate payoff over gathering new information. Crucially, beyond this general distinction, computational modeling (i.e., algorithmic representations of the observed data to analyze how children explore different options) has differentiated between two distinct types of exploration strategies: random versus directed exploration (e.g., Schulz et al., 2019; Wilson et al., 2021). Random exploration involves the haphazard selection of options without a systematic strategy (Nussenbaum & Hartley, 2019). In contrast, directed exploration involves using focused, systematic sampling strategies that progressively reduce uncertainty in options. While directed exploration represents an information-prioritizing strategy often seen in supportive, stable environments, random exploration represents a “noisier” search strategy that may function as a form of risk-taking.

As highlighted by prior literature (e.g., Blanco & Sloutsky, 2024; Frankenhuis & Gopnik, 2023; Gopnik, 2020), human development follows a general developmental trajectory regarding the exploration–exploitation trade-off: childhood functions as a protected period of broad, variable exploration, which gradually transitions into exploitation as the individual matures and assumes adult responsibilities. This transition allows children to build a rich model of the world before they need to act on efficiency.

Yet, the normative trajectory is sensitive to the environmental inputs. The balance of exploration and exploitation is theorized to be a product of hyperparameters shaped by early experiences (e.g., early adversity; Frankenhuis & Gopnik, 2023; Nussenbaum & Hartley, 2024). According to life-history models, biases toward exploitation and heightened random exploration might align with a fast life-history strategy (Belsky et al., 1991; Del Giudice & Haltigan, 2023; Ellis et al., 2022; Frankenhuis & Gopnik, 2023). Within this evolutionary framework, adversity is conceptualized as environments characterized by high extrinsic morbidity and mortality. These survival risks are frequently embedded within poverty and socioeconomic disadvantages, which often encompass both chronic resource deprivation and environmental threat, and unpredictability. In these adverse contexts, extended exploration becomes costly and potentially dangerous as extended exploration may never pay off if an individual’s survival is compromised, thereby favoring exploitation. In addition, while a shift toward exploitation allows individuals to secure immediate rewards in poverty-related adverse environments, heightened random exploration might function as another hallmark of the “fast” strategy for several reasons. First, directed exploration requires “high-cost” cognitive investment (e.g., sustained attention, working memory, and systematic planning) that may not yield sufficient returns in adverse environments. In contrast, random exploration may function as a “low cost, low effort” search strategy. Second, life-history theory highlights impulsivity and reduced inhibitory control as key components of a faster life-history strategy (Belsky et al., 1991; Del Giudice & Haltigan, 2023; Ellis et al., 2022); computationally, this tendency may manifest as “noisier” or “high-temperature” search behavior (i.e., random exploration). Finally, adversity-related changes in reward neural circuitry (e.g., impaired functional activity in reward circuit, altered neural connectivity [e.g., striatum to prefrontal cortex]; smaller volume in hippocampus and prefrontal cortex, Hanson et al., 2021; Johnson et al., 2016; Herzberg & Gunnar, 2020) may limit a child’s ability to effectively integrate feedback during the tasks, which can be further manifested as random rather than directed exploration patterns. As such, our goal in the present study is to explore children’s balancing of exploration and exploitation as sequelae of their family experiences and their relation to children’s social and behavioral functioning.

Considering the two different types of exploration (i.e., random vs. directed exploration) and exploitation together, children may vary in how they employ the three key components (Harms et al., 2024; Meder et al., 2021; Schulz et al., 2019). Despite this, prior literature has predominantly adopted a variable-centered approach by focusing on one of the three indicators at a time within the entire sample, which limits our understanding of children’s exploration–exploitation strategies holistically (i.e., by considering all three indicators simultaneously to understand each person’s distinct strategies). To address this limitation, we employed a person-centered approach to examine subgroups based on multiple indicators of exploration versus exploitation, seeking to parsimoniously summarize multiple characteristics via profile membership and identify unique configurations of key exploration–exploitation traits.

### Family precursors of exploration–exploitation profiles

According to theory and prior literature, exposure to contextual adversity might accelerate the life-history strategy, which may be manifested in two ways in the exploration–exploitation strategies children use. First, exposure to contextual adversity may accelerate the life-history strategy that shifts toward exploitation, prioritizing the acquisition of immediate rewards (Frankenhuis & Gopnik, 2023). This accelerated shift aligns with theoretical and research findings on greater present orientation (i.e., reduced future discounting) in individuals exposed to adversity (e.g., Frankenhuis et al., 2016). Existing research has documented links between exposure to adversity (e.g., physical abuse, neglect) and reduced exploration and heightened exploitation in adults and children (e.g., Davies et al., 2022; Humphreys et al., 2015; Lenow et al., 2017; LIoyd et al., 2022; Xu et al., 2023). For example, Lenow et al. (2017) and Lloyd et al. (2022) adopted a foraging paradigm and found that adults with greater childhood adversity (e.g., neglect) or who experienced stress (e.g., chronic stress) showed under-exploration and over-exploitation in the foraging task.

Second, as discussed before, prior research has also documented that individuals exposed to contextual adversity may show heightened random exploration, in contrast to the directed, information-seeking exploration. For instance, Hanson et al. (2017) reported that adolescents who experienced childhood physical abuse were more likely to exhibit random exploration and less likely to use information acquired during decision-making in a probability-learning task in which they learned about different reward probabilities behind two paired random objects. Findings from Xu and colleagues (2023) in a horizon task indicated that youth with unpredictable childhood experiences (e.g., inconsistent and unpredictable parental involvement, financial insecurity) were less likely to select and value the direct exploration approach of reducing uncertainty by selecting high-information options.

While we draw upon this prior literature, it is important to note significant methodological heterogeneity in how exploration and exploitation are operationalized across different cognitive tasks and types of adversity. Despite these differences, the literature converges on the broad conclusion that exposure to adversity may alter the exploration–exploitation balance. Notably, despite the crucial contributions, this line of work has largely adopted a variable-centered approach, evaluating the linear associations between adversity and one indicator of exploration–exploitation strategy at a time. Building on this research, the present study focuses on one common form of family adversity (i.e., socioeconomic risks) as a precursor to exploration–exploitation profiles.

### The relation between psychological functioning and exploration–exploitation profiles

Our final goal was to examine relations between children’s exploration–exploitation profiles and their social and behavioral adjustment. As established earlier, biases toward exploitation *and heightened* random exploration are theorized to be part of a fast life strategy (Frankenhuis & Gopnik, 2023). Therefore, it stands to reason that preferences for exploitation and random exploration should be correlated with other fast life strategy indicators including externalizing problems, attention difficulties, and opportunistic interpersonal orientations that reflect poor social competence (Doom et al., 2016; Li et al., 2018). Notably, we consider social competence within the life-history framework, with greater social competence aligning with a slow life-history strategy. This is because social competence involves regulating one’s behavior to optimize social relationships (Taborsky & Oliveira, 2012), and the tendency to compromise immediate advantages toward forming long-term, mutually beneficial social relationships. In contrast, poor social competence has been conceptualized as an indicator of a fast life-history strategy, marked by opportunistic advantage-taking interpersonal orientations (Belsky et al., 1991; Doom et al., 2016; Li et al., 2018). Thus, individuals adopting a fast life-history strategy within social relationships may prioritize immediate opportunistic gains over the regulation required for forming long-term, mutually beneficial relationships.

There has been only one study, to our knowledge, that directly addressed this question. Somerville et al. (2017) found that adolescents’ preferences for random exploration were associated with greater self-reported risk-taking behavior. In contrast, no association was found between risk-taking behavior and directed exploration (i.e., preferences for high-information option). Given the limited research in this area and the call in Frankenhuis and Gopnik (2023), we examine children’s psychological sequelae of exploration–exploitation profiles.

### The present study

Our multi-method longitudinal study aims to identify family socioeconomic antecedents and child psychological sequalae of exploration–exploitation profiles drawn from computational modeling. We propose that children who adopt a faster life-history strategy may manifest two types of exploration–exploitation strategies, which may be characterized by either (a) heightened exploitation that prioritizes seizing immediate rewards (e.g., Lenow et al., 2017; LIoyd et al., 2022), and/or (b) heightened random exploration (e.g., Hanson et al., 2017; Xu et al., 2023). Yet, given the limited and inconsistent evidence so far, these hypotheses are tentative. In turn, children exhibiting exploration–exploitation patterns that align with the fast life-history strategy are also more likely to experience greater socioeconomic risks and manifest more externalizing problems, attention problems, and lower social competence. Notably, as we situate our study within the life-history framework, individuals are theorized to adapt their strategies according to the environmental cues. Whereas prior work has focused on various types of adversity (e.g., maltreatment, chronic stress, unpredictability), here we focus on socioeconomic risks, which are powerful and broad indicators of environmental risk that often embed harshness, resource scarcity, and chronic instability within the family context (e.g., Belsky et al., 2012; Skinner et al., 2023). Furthermore, the present study utilized trial-by-trial data and builds on our prior work (Davies et al., 2022) in the following ways. First, prior work utilized behavioral data and focused on children’s implicit-learning performance (i.e., the times choosing the tree with the highest winning probabilities). We employed computational modeling in uncovering different exploration mechanisms (i.e., directed vs. random exploration), in addition to game performances. Second, in addition to examining the contextual antecedents, we also explore the developmental implications of exploration–exploitation profiles as promoted in Frankenhuis and Gopnik (2023).

## Method

### Participants

Participants were 243 families with a preschooler recruited in a mid-sized city in the Northeastern part of the United States. Families were recruited through universal pre-K programs, Head Start Agencies, and childcare providers. All mother–child dyads were living together at the first measurement occasion, and children were on average 4.60 years (*SD* = 0.44) at Wave 1 (56% children were girls). Children were racially and ethnically diverse, with 46% children identified as Black or African American, followed by 39% White, and 13% as multiracial, or another race (2%). In addition, 19% of the children were Latinx. The average household earned income was $31,231 (range = $2,000 to $121,000), with the majority of the families receiving public assistance (70%). The median level of parental education was a GED or high school diploma (See additional demographic characteristics of the sample in Supplemental Material, Table S1). Retention rate was 91% across the two measurement occasions that were spaced two years apart. At Wave 3, children were on average 6.77 years (*SD* = 0.48). At each wave, parents and children completed two visits – scheduled roughly one week apart – at a research laboratory. Because this study used data from the large longitudinal project designed to examine interparental relationships and children’s emotional security, the sample size was determined during the project’s initial design stage based on power analyses for its primary research goals. Consequently, we did not perform a priori power analyses for the present research goals. The study is not pre-registered.

### Ethical consideration

The protocol of this project was approved by the Institutional Review Board of the University of Rochester (Title of the study: Children’s Development in the Family, case number: 00030261). Informed consent was obtained from both parents at each wave. Parents and teachers were compensated monetarily for their participation, and children received small toys.

### Measures

#### Family socioeconomic predictors (Wave 1)

To measure socioeconomic resources of the family, we assessed two distinct sources of family income from a maternal demographic interview at Wave 1. Earned (annual) household income consisted of household income in the form of employment salaries, wages, and overtime pay in units of $1,000. As shown in Table 1, values ranged from 0 (*N* = 58) to 121.0. Given that the distribution did not exhibit excessive skewness (0.79) or kurtosis (−0.25), we utilized the raw earned income values without further transformation. In addition, given that earned income, as an indicator of family socioeconomic resources, may be confounded by the family size, we performed sensitivity analyses (See details in Results) evaluating the earned family income adjusted for the family size (i.e., adjusted earned income = total earned income/the sum number of adults and children living in the household; see distribution of the family socioeconomic predictors in Supplemental Material, Figure S2).

**Table 1.** Descriptive information for the primary study variables Table 1 long description.

	1	2	3	4	5	6	7	8
1. Uncertainty-directed exploration (β)	–							
2. Random exploration (τ)	−.18**	–						
3. Total rewards obtained	−.16*	−.46**	–					
4. Public assistance	−.22**	.14*	−.01	–				
5. Earned income	.20**	−.04	−.03	−.66**	–			
6. Externalizing symptoms	−.20*	.11	−.09	.23**	−.32**	–		
7. ADHD symptoms	−.21**	.19*	−.07	.22**	−.30**	.71**	–	
8. Social competence	.13†	−.14†	.14†	−.17*	.26**	-.72**	−.71**	–
*N*	214	214	214	241	241	175	175	175
*Mean*	12.15	4.44	43.59	9.86	31.23	0.24	0.50	−0.003
*SD*	8.70	6.17	7.54	10.24	28.94	0.36	0.52	0.90
*Min*	0	0.001	27.00	0	0	0	0	−2.88
*Max*	20	20	77.00	54.80	121.00	1.70	2	1.12
Skewness	−0.38	1.88	0.92	0.99	0.79	1.92	1.12	−1.07
Kurtosis	−1.67	2.07	2.65	0.75	−0.25	3.47	0.35	0.80

†*p* < .1, *p < .05, **p < .01.

Public assistance was indexed by income (in units of $1,000) derived from governmental programs designed to provide financial aid and support to families in need (e.g., Temporary Assistance for Needy Families, TANF). As shown in Table 1, scores ranged from 0 (*N* = 73) to 54.80. We treated zero values as valid responses indicating the absence of public assistance than missing data or unwillingness to report income. Once again, because the distribution fell within acceptable limits for skewness (0.99) and kurtosis (0.75), no further transformations were performed. Crucially, because public assistance programs in the United States (e.g., TANF) allocate funds using formulas that explicitly account for household size relative to family gross income, this measure inherently reflects the level of financial strain adjusted for family size. As such, our sensitivity analyses for public assistance did not further adjust for family size.

Notably, earned income and public assistance represent distinct sources of financial support that reflect different aspects of socioeconomic resources, and thus, the inclusion of both allows for a more comprehensive assessment of the family’s socioeconomic context, capturing both economic capacity and need-based support.

#### Reward probability task (Wave 3)

A more detailed description of the reward probability task, an adaptation for 4–6-year-olds that has been used in this age group, can be found in Davies et al. (2022); original task: Starling et al. (2018). Notably, the task was administered at the third wave, which is two years following the initial measurement occasion (Wave 3 Mean child age = 6.77 years). The task consisted of a 120-trial force-choice task (See a graphic illustration of the task in Supplemental Material, Figure S1), during which children used a touchscreen and selected one out of three trees, guiding a bird to recover a hidden reward (i.e., a cherry). Feedback was provided immediately after each trial, such that correct trials were indicated by the bird appearing in the tree with a happy expression and cherries, and an increase in the orange power bar denoting an increase in the bird’s strength. In contrast, following an incorrect choice, the bird will appear next to the incorrect tree with a sad expression and a red cross, in addition to a decrease in the bird’s power bar. The task consisted of six 20-trial blocks, and there was a majority tree containing cherries for 70% of the trials, with the other two trees each containing cherries for 15% of the trials (i.e., 70%, 15%, 15%). The majority tree was fixed at tree #3 for the first three blocks (i.e., 60 trials), and then, following a brief mid-task break, the majority tree switched to tree #1 without warning for the second 60 trials. The location of the reward was pseudorandomized to ensure a relatively equitable distribution of reward across all six blocks (See more details in the Supporting information). Similar to prior work (Davies et al., 2022), we assessed performance on the task by the total rewards acquired during the game. However, in complementing the previous assessment approach (Davies et al., 2022), we adopted a computational modeling approach to model children’s trial-by-trial selection over the whole trajectory to investigate the underlying exploration strategies and develop person-based profiles (see details in data analysis & results).

#### Child socioemotional functioning (Wave 3)

At Wave 3, elementary school teachers completed scales from the well-established MacArthur Health and Behavior Questionnaire (HBQ, Albow et al., 1999) to assess their psychological adjustment. Three indicators were selected based on their relevance to life-history strategies. Whereas Externalizing Symptoms Scale consisted of the average of 30 items indexing oppositionality, over hostility, relational aggression, and conduct problems (e.g., “physically attacks people; *α* =.96), the attention deficits and hyperactivity symptoms (ADHD) scale assessed inattention and impulsivity (15 items; “impulsive or acts without thinking,” *α* = .94). Response alternatives were: 0 = Never/Not true; 1 = Sometimes or somewhat true; 2 = Often or very true. The third assessment was a Social Competence composite consisting of the Peer Acceptance (8 items; e.g., “is liked by other children who seek him/her for play”) and Prosocial Behavior (20 items; e.g., “is considerate of others’ feelings”). Because the response alternatives on the two scales differed (i.e., Peer Acceptance ranged from 1 = *Not at all like* to 4 = *Very much like*; Prosocial Behavior ranged from 0 = *Rarely applies* to 2 = *Certainly applies*), the scales were standardized and averaged together to form the Social Competence composite (item-level *α* = .96).

## Data analyses and results

Data analyses proceeded in two stages: (a) computational modeling of children’s choices to derive parameters of uncertainty-directed exploration, random exploration, and total rewards during the reward probability task; and (b) identification of profiles of the children’s exploration–exploitation strategies via latent-profile analyses. Codes for the parameter estimation and model fitting can be found in the following repository (https://osf.io/pfxac/overview).

### Computational modeling

Following Schulz et al. (2019) and Meder et al. (2021), we used Bayesian functioning to adaptively learn and map a location option (i.e., out of the three tress) with rewards. Our computational model involved the sampling strategy and the choice rule as described in Meder et al. (2021). Importantly, although some of the prior computational modeling studies utilized older samples (e.g., adolescents; Hanson et al., 2017; Xu et al., 2023), our task was appropriate for the current age group, and the same computational modeling strategy (i.e., Bayesian UCB) and the specific model parameters derived from the models have been successfully used in this age group (e.g., 4–9-year-olds; Meder et al., 2021). More specifically, this process generates equal prior beliefs for reward of any of the three locations (i.e., for binary rewards, the common Beta distribution was set up as the prior for all three tree locations) and uses the Bayesian Upper Confidence Bound (Bayesian UCB) sampling strategy to map the beliefs into valuations of each option at a given time. For tree *i* at time *t*, Bayesian UCB is formulated as the weighted sum function:

$$BUCB_{i}\left(t\right)=u_{i}\left(t\right)+\beta \sigma_{i}(t)$$

In the Bayesian UCB function, each location has a posterior mean expected reward given the data observed up to time *t*, *u*_*i*_(*t*), and is associated with a level of uncertainty given the data observed up to time t, *σ*_*i*_(*t*) (i.e., measured via the standard deviation, with a larger standard deviation indicating greater uncertainty). The Bayesian UCB approach is potentially a more accurate measure of uncertainty by incorporating both prior information and all the observed data via Bayesian updating.

Notably, the parameter *β* represents the extent to which the uncertainty is valued in the UCB function, such that higher *β* values indicate a strong preference toward exploring options that have greater uncertainty, and a lower *β* value approaching zero reflects that decisions were dominated by the expected reward regardless of the uncertainty. Therefore, the UCB sampling strategy offers an opportunity to balance exploration (i.e., exploring more uncertain options) versus exploitation (i.e., exploiting a higher expected reward value).

As an illustration of the sampling strategy, consider two options: Option one has an expected reward *u*_1_(*t*) = 10 and *σ*_1_(*t*) = 5, whereas Option two has an expected reward of *u*_2_(*t*) = 5 and *σ*_2_(*t*) = 10. Thus, option one had a higher expected reward than option two, but option two was more uncertain. Thus, for example, if *β* = 2 (i.e., an example of a higher weight given to exploring uncertain option), then UCB (Option 1| *β* = 2) = 20, and UCB (Option 2| *β* = 2) = 25, which favors option 2. Thus, higher *β* reflects a greater preference for exploring the more uncertain option 2. In contrast, if *β* = 0.5 (i.e., an example of a lower weight given to exploring uncertain option), then UCB (Option 1| *β* = 0.5) = 12.5 and UCB (Option 2| *β* = 0.5) = 10, favoring to exploit the option (i.e., option 1) with a higher expected reward. Thus, by estimating the individual-specific *β* weight based on the child’s search behavior, we captured the child’s tendency for uncertainty-directed exploration.

Similar to Schulz et al. (2019) and Meder et al. (2021), we used a SoftMax function to map the UCB values into choice probabilities:

$$p\left(x\right)=\frac{\exp\;\left(BUCB_{i}\left(t\right)/\tau \right)}{\sum_{j=1}^{N}\exp\left(\frac{BUCB_{j}\left(t\right)}{\tau }\right)}$$

This formulates the probability to select each tree location by the exponential of B-UCB value for each tree location (*i*) divided by the sum exponential B-UCB values for all tree-locations (*j:* tree 1 to *N*), both divided by the temperature parameter *τ*. The temperature parameter *τ* thus represents the randomness in the choices given the existing B-UCB values, such that a lower τ (i.e., low randomness) means that the probability of choosing a certain option is heavily concentrated to the B-UCB value (i.e., more likely to select the option with a higher B-UCB value), and a really large τ (e.g., approaching positive infinity) would mean that all options will have equal probability of being chosen regardless of the B-UCB values. Once again, we estimate a person-specific τ value based on the child’s behavior during the task, with a higher τ indicating more random exploration.

**Figure 1.**
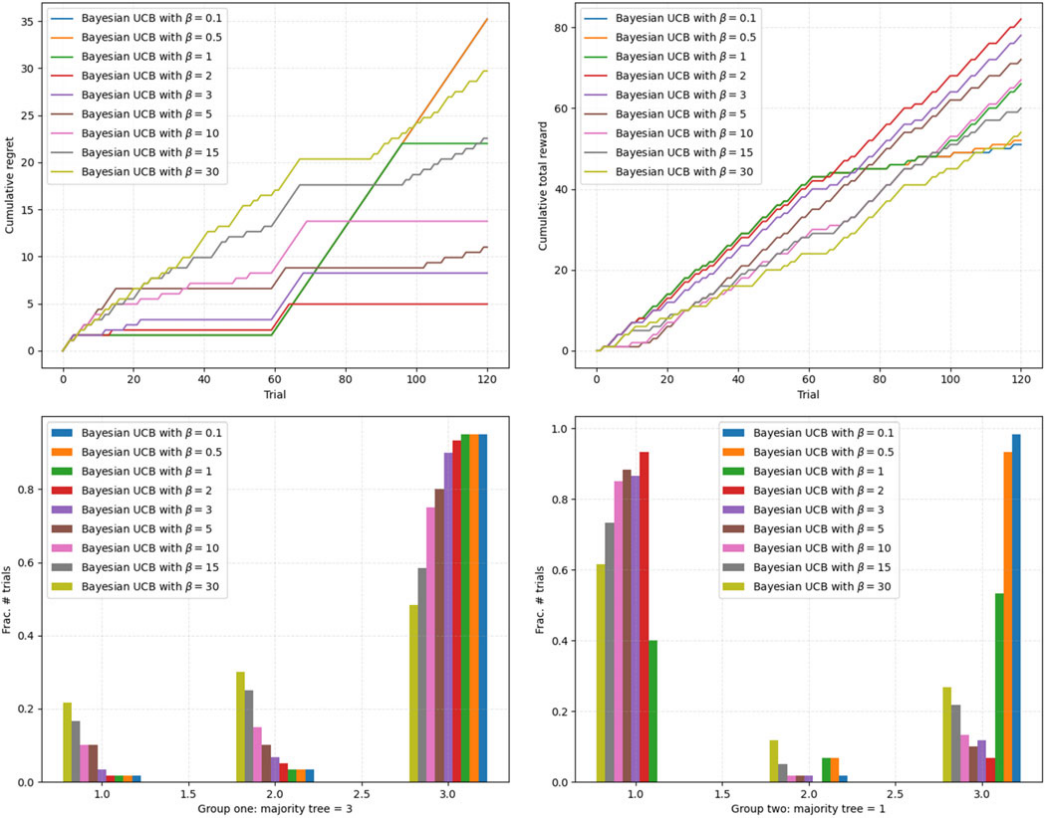
Figure 1 long description. Simulated data with different levels of uncertainty-directed exploration (i.e., *β* values).

As an additional note, the present task involved a switch of majority tree at the second half of the game (i.e., in the first 60 trials, majority tree = Tree location #3, and in the second 60 trials, majority tree = Tree location #1). We performed simulations with different uncertainty-directed exploration (i.e., *β* values ranging from 0.1 to 30) to illustrate the performance of learning under each condition. As shown in Figure 1, all simulated scenarios seemed to be able to learn the majority tree fairly well (i.e., by figuring out the optimal tree at the first half of the task). Lower uncertainty-directed exploration (i.e., lower *β*) seemed to exploit more with a greater likelihood of selecting the optimal tree, whereas higher uncertainty-directed exploration (i.e., higher *β*) seemed to be more diverse in their options (i.e., by selecting the other two options at times). A high level of uncertainty-directed exploration might dampen the overall performance (i.e., by not always selecting the majority tree in favor of gaining information). Notably, although the strategies with strong exploitation tendencies (e.g., *β* = 0.5) exhibited great performance at the beginning, these strategies might be too exploitive to learn about the new majority tree after the switch in the second half of the game. Thus, strategies that yielded the highest reward overall seemed to achieve a balance between exploitation and exploration, such that it can efficiently learn the optimal tree and exploit the rewards but also explore enough to learn and adapt when there is a change in the environment (i.e., switch of the optimal tree).

We performed all the modeling via Python, with the input being child’s trial-by-trial choice, and output being the parameters described above (i.e., *β*, *τ*, and total rewards obtained during the entire game). For numeric stability (e.g., too high of *β* and *τ* values might lead to almost completely random choices in the strategies given we had a fairly simple game setting), we set up maximum boundary values of *β* and *τ* at 20. As shown in Table 1, all three parameters were correlated at a low to moderate level, but in opposite directions. More specifically, greater uncertainty-directed exploration (*β*) was associated with lower random exploration (*τ*). Both greater uncertainty-directed exploration (*β*) and random exploration (*τ*) were linked to lower total rewards obtained during the task.

### Person-centered analyses for child exploration patterns

Data analyses for the second stage were all conducted in Mplus 8.2 (Muthén & Muthén, 1998-2017). This stage involved investigating the subgroups of child exploration–exploitation based on the parameters derived from the computational analyses through latent profile analysis (LPA) and examining the environmental antecedents and child socioemotional functioning associated with the latent profiles.

Our LPA involved three parameters derived from computational modeling: directed exploration (*β*), random exploration (*τ*), and total rewards obtained (i.e., an indicator for how effective children’s search strategies were). LPA allows for examining the interdependence in multiple observed characteristics (e.g., directed exploration, random exploration in the present study) through latent-profile membership (e.g., Lanza & Rhoades, 2013). As a result, it is more parsimonious than evaluating numerous interactions among the indicators (i.e., with three indicators, at least one three-way interaction, and three two-way interactions to be tested). In addition, LPA allows the researchers to draw on both theory and data-driven model comparison results to select the optimal latent profile solution. We ran LPA models with one to four profiles while considering the theory and interpretability of the profiles using several model fit indices to select a solution. As one subset of fit indices, lower Akaike information criteria (AIC), Bayesian information criteria (BIC), and adjusted BIC indicate greater model fit. In addition, greater entropy values (particularly values higher than 0.80), suggest sufficiently accurate classification. Finally, significance in Lo–Mendell–Rubin (LMR), Vuong–Lo–Mendell–Rubin (VLMR), and bootstrapped LMR tests indicate a superiority of k-class than k-1-class models (Lo et al., 2001).

**Figure 2.**
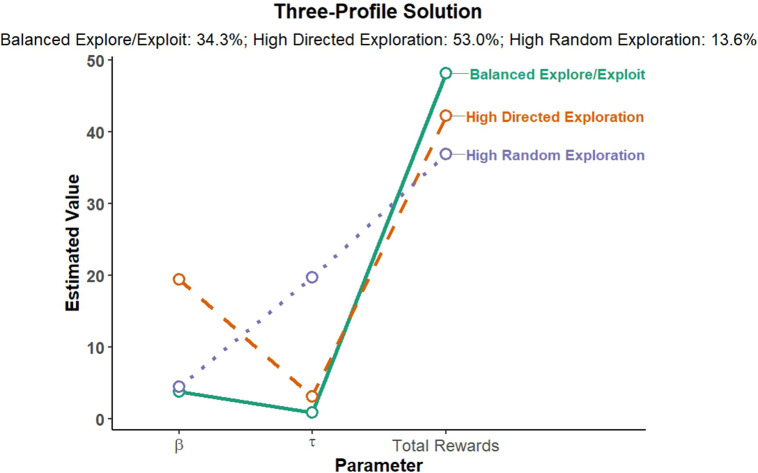
Figure 2 long description. The three-profile solution for child exploration patterns. *Note*. The horizontal axis plotted in order: Uncertainty-directed exploration (beta), Random exploration (tau), and total rewards obtained during the game.

As shown in Table 2, we selected the three-profile model as optimal solution based on the conceptual meaning and the interpretability of its identified profiles and the superior model fit indices overall (also see additional details in Supporting information). The patterns of the three-profile solution are presented in Figure 2 (See Supporting information, Table S3). The horizontal axis plotted the uncertainty-directed exploration (*β*), random exploration (*τ*), and the total rewards, the profile obtained highest rewards (i.e., Green solid line) consisted of 34.3% children. Children in this “*Balanced Exploration/Exploitation*” profile appeared to demonstrate fairly “optimal” strategy in which they showed (a) the lowest random exploration; (b) the sufficient level of uncertainty-directed exploration to learn about the task (i.e., the location of the majority tree, and the switch in majority tree in the second half of the task); (c) limited uncertainty-directed exploration (i.e., not too much to undermine their performance); and (d) high acquisition of rewards. Next, the profile obtained the second to the highest rewards (i.e., Red broken line) consisted of 53.0% children. This group exhibited the highest uncertainty-directed exploration, low random exploration, and obtained a high amount of reward. Accordingly, we labeled this profile of children as the “*High Directed Exploration*” profile. Finally, the profile exhibiting medium uncertainty-directed exploration, the highest random exploration, and the least rewards obtained (i.e., the Purple dotted line) involved 13.6% of children and was labeled as the “*High Random Exploration*” profile.

**Table 2.** Fit indices for latent profile solution (*N* = 214) Table 2 long description.

	1-Profile	2-Profile	3-Profile	4-Profile
AIC	4,400.17	4,044.11	3,883.26	Did not converge (See details in supplemental material)
BIC	4,420.37	4,077.77	3,930.39	
ABIC	4,401.36	4,046.9	3,886.02	
Entropy	–	.99	.97	
LMR	–	.00	.10	
VLMR	–	.00	.09	
B-LMR	–	.00	.00	
% Class 1	214 (100%)	186 (86.92%)	73 (34.11%)	
% Class 2	–	28 (13.08%)	114 (53.27%)	
% Class 3	–	–	27 (12.62%)	
% Class 4	–	–	–	

### Environmental antecedents and child socioemotional functioning associated with exploration patterns

To examine how family context was linked to child exploration profiles, we employed the Vermunt’s three-step approach (Vermunt, 2010), which carries out multinomial logistic regression predicting the latent-profile membership without changing the profile solutions. As such, we included the two indicators of family socioeconomic status (i.e., parent earned income, total public assistance received) as predictors of the latent profiles. As shown in Table 3, earned income was not linked to any latent profiles, but the amount of public assistance received by the family was related to the profiles. Greater public assistance was significantly linked to lower likelihood for children to be classified into the High Directed Exploration profile compared to the High Random Exploration Profile. We performed several follow-up sensitivity tests to examine the robustness of these findings. First, we re-ran the model evaluating the role of public assistance and parent earned income while controlling for additional demographic covariates (i.e., child age in months at Wave 3 given child completed the reward probability task at Wave 3, and child race). Our findings remained the same (Supplemental Material, Table S4), such that greater public assistance received by the family was still significantly linked to lower likelihood for children to be categorized into the high-directed exploration compared to the high-random exploration profile. Second, as mentioned before in the methods, we performed the second sensitivity analysis using socioeconomic indicators adjusted for family size. In particular, we included earned income adjusted for family size but retained the original public assistance income (given it inherently reflected financial strain accounted for family size) as alternative predictors associated with latent profile memberships. Findings (see Supplemental Material, Table S2) again remain the same, such that receiving greater public assistance was still linked to a significantly lower likelihood for children to be categorized into the higher-directed exploration profiles compared to the high-random exploration profile. In addition, the earned income adjusted for family size was still not significantly linked to profile memberships. Third, although parent earned income and public assistance represent theoretically distinct facets of socioeconomic risk, we additionally applied a post hoc Bonferroni correction (*α* = 0.05/2 = 0.025) across these two predictors as a highly conservative sensitivity test of robustness for our contextual findings. Our findings remained significant after the correction, such that children receiving greater public assistance were still more likely to be categorized into the High Random Exploration profile than into the High Directed Exploration profile (i.e., the *p*-value of .02 fell below the adjusted alpha threshold of .025).

**Table 3.** Logistic regression coefficients for three-class solution (*N* = 214) Table 3 long description.

		*Coefficient (SE)*	*Z*	*p*
**Profile 3 (high-random exploration) as comparison class**				
Profile 1 (balanced exploration/exploitation)	Earned income	−0.01 (0.01)	−1.26	.21
	Public assistances	−0.05 (0.03)	−1.79	.07
Profile 2 (high-directed exploration)	Earned income	−0.003 (0.01)	−0.27	.78
	Public assistances	−0.06 (0.03)	−2.37	.02
**Profile 1 (balanced exploration/exploitation) as comparison class**				
Profile 2 (high-directed exploration)	Earned income	0.01(0.01)	1.48	.14
	Public assistances	−0.02(0.02)	−0.86	.39

**Figure 3.**
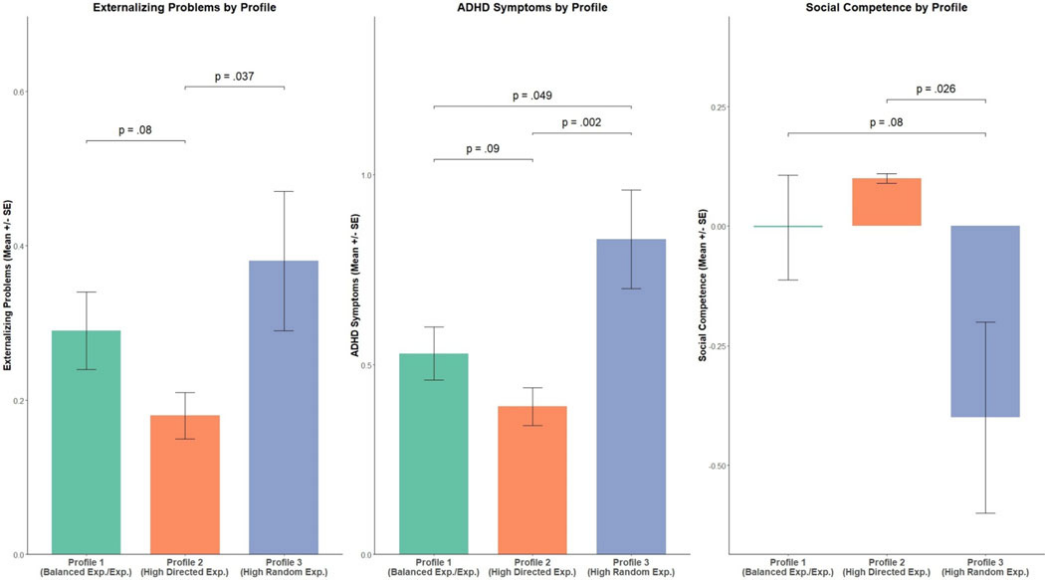
Figure 3 long description. Exploration patterns and child socioemotional functioning (*N* = 214). *Note.* Profile 1 (Green bar): Balanced Exploration/Exploitation Profile; Profile 2 (Orange bar): High Directed Exploration Profile; Profile 3 (Purple bar): High Random Exploration Profile.

Finally, we adopted the Bolck–Croon–Hagenaars method (BCH method, Bolck et al., 2004) to examine how child exploration–exploitation profiles were concurrently related to child socioemotional functioning. The BCH methods included the socioemotional functioning as outcome associated with the latent profiles, and carries out omnibus tests among all profiles and then follows significant omnibus tests by pairwise comparisons between profiles. Because our three socioemotional outcomes (Externalizing Symptoms, ADHD Symptoms, and Social Competence) were chosen a priori based on their specific theoretical relevance to life-history strategies, we did not apply a family-wise error correction (e.g., Bonferroni) across the three distinct outcome models. Instead, Type I error was managed within each functional domain by the prerequisite of a significant omnibus test in BCH method prior to evaluating pairwise comparisons. As shown in Table 4, omnibus tests indicated significant differences among the three profiles in externalizing problems and ADHD symptoms. Pairwise comparison (Table 4 and Figure 3) indicated significant differences in externalizing problems and ADHD symptoms between the High-directed Exploration versus the High-Random Exploration profile. The High-Random Exploration profile specifically exhibited higher externalizing and ADHD symptoms than the High-Directed Exploration profile. In addition, the High-Random Exploration profile also demonstrated significantly more ADHD symptoms compared to the Balanced Exploration/Exploitation profile. Finally, comparisons of the Balanced Exploration/Exploitation and High Directed Exploration profiles did not yield significant differences.

**Table 4.** Exploration patterns and child socioemotional functioning (*N* = 214) Table 4 long description.

	Profile 1 (balanced exploration/exploitation)	Profile 2 (high-directed exploration)	Profile 3 (high-random exploration)	Mean (*SE*)
**Externalizing problems**				
Profile 1 (balanced exploration/exploitation)	–			0.29 (0.05)
Profile 2 (high-directed exploration)	*X* ^2^ = 3.07, *p* = .08 Marginally, 1 > 2	–		0.18 (0.03)
Profile 3 (high-random exploration)	*X* ^2^ = 0.74, *p* = .39	*X* ^2^ = 4.33, *p* = .037 3 > 2	–	0.38 (0.09)
Overall test	*X* ^2^ = 6.20, *p* = .045			
**ADHD symptoms**				
Profile 1 (balanced Exploration/Exploitation)	–			0.53 (0.07)
Profile 2 (high-directed exploration)	*X* ^2^ = 2.89, *p* = .09 Marginally, 1 > 2	–		0.39 (0.05)
Profile 3 (high-random exploration)	*X* ^2^ = 3.86, *p* = .049 3 > 1	*X* ^2^ = 9.42, *p* = .002 3 > 2	–	0.83 (0.13)
Overall test	*X* ^2^ = 10.59, *p* = .005			
**Social competence**				
Profile 1 (balanced exploration/exploitation)	–			−0.003 (0.11)^a^
Profile 2 (high-directed exploration)	*X* ^2^ = 0.49, *p* = .49	–		0.10 (0.10)
Profile 3 (high-random exploration)	*X* ^2^ = 3.03, *p* = .08 Marginally, 3 < 1	*X* ^2^ = 4.95, *p* = .026 2 > 3	–	−0.40 (0.20)
Overall test	*X* ^2^ = 4.95, *p* = .08			

^a^Child social competence was created by averaging the standardized peer acceptance and prosocial behavior; thus a positive value indicates greater than sample mean, and a negative value indicates lower than sample mean values.

## Discussion

This multi-method longitudinal study applied computational modeling and LPA to characterize the nature, family precursors, and psychological adjustment correlates of children’s exploration–exploitation strategies during a reward probability task. Findings revealed three profiles: a balanced exploration/exploitation profile, a high-directed exploration profile, and a high-random exploration profile. Higher levels of public assistance received by the family, a proxy for economic strain, was linked to greater likelihood for children to be classified into the high-random exploration profile. With regard to child functioning, the high-random exploration profile seemed to exhibit higher externalizing problems and ADHD symptoms.

LPAs yielded three exploration–exploitation profiles. Inspecting the profiles and the modest-to-moderate bivariate correlations among the profile indicators support the premise that uncertainty-directed exploration, random exploration, and the total rewards are relatively distinct dimensions. Notably, the high-random exploration profile seemed to exhibit the most haphazard exploration and obtained the lowest amount of reward. This profile of children also seemed to be raised in families receiving more public assistance (than the high-directed exploration profile). Crucially, this association remains statistically robust in multiple sensitivity analyses, and even when applying a conservative Bonferroni correction for multiple testing across our two socioeconomic indicators. These findings align with prior work, such that children who experienced greater adversity (i.e., physical abuse) showed more random exploration (i.e., Hanson et al., 2017). It is noteworthy that family public assistance, rather than earned income, was the antecedent for a higher likelihood of membership into the random exploration. While these two sources of income are related, they may represent distinct aspects of the family’s socioeconomic position: the earned income reflects current financial resources and capacity, public assistance often serves as a proxy for financial strain. Our findings suggest that the presence of high financial strain and economic risk is a more salient predictor of children’s random exploration strategy. Although future work is needed to better understand this association, it is possible that public assistance serves as a more robust indicator of a confluence of adversity, including impoverishment, discrimination, and inequities in education and healthcare (Santiago et al., 2011; Weitoft et al., 2008).

The high-random exploration profile exhibited the highest ADHD symptoms and greater externalizing problems (relative to the high-directed exploration profile). Although we did not directly measure risk-taking behavior, this finding is consistent with previous work linking random exploration and greater risk-taking behavior (Somerville et al., 2017). We interpret this consistency from the life-history perspective: the outcome we measured (e.g., higher externalizing problems and ADHD symptoms) is conceptually and empirically related to traditional risk-taking behavior (Belsky et al., 1991; Doom et al., 2016; Li et al., 2018; Thompson et al., 2011), as they all reflect a “riskier” life-history strategy. For additional clarification, when we describe this profile as “risky,” we refer to its alignment with the riskier end of the life-history strategy, rather than directly referring to the actual “risk-taking behavior.”

The pattern of findings for the high-random exploration profile may reflect several underlying processes. First, the coupling of haphazard search methods and low acquisition of reward in the high-random exploration profile might signify impaired reward sensitivity (i.e., impaired ability to incorporate the reward feedback in guiding the exploration). In supporting this interpretation, studies have repeatedly shown that exposure to childhood adversity is associated with reduced responsiveness to reward (e.g., Dennison et al., 2019; McLaughlin et al., 2019; Oltean et al., 2023; Sheridan et al., 2018). Second, effective strategies during the reward probability task involve several cognitive skills, including allocating and sustaining attention, retaining information on past winning probabilities and uncertainty with the reward stimuli in current trials (i.e., working memory), and inhibiting reflective impulses to randomly select different options (i.e., inhibitory control). Thus, in accord with life-history theory, the relation between higher socioeconomic risk and children’s random exploration strategies might be part of a shift away from investment in slow-life capacities and toward a risky fast life orientation (i.e., reduced executive functions and focused attention, greater externalizing problems Blair et al., 2011; Finegood & Blair, 2017). Third, exposure to environmental risks might alter neural development in brain regions and connectivity related to executive functions and reward processing (e.g., impaired functional activity in reward circuit & altered neural connectivity; e.g., Hanson et al., 2021; Herzberg & Gunnar, 2020; Johnson et al., 2016), which may account for the relation between exposure to early adversity (e.g., socioeconomic risks) and heightened random exploration and risky behavior as described above (e.g., greater externalizing problems).

Children in the high direct exploration profile experienced relatively lower socioeconomic risks and exhibited lower levels of risky behavior, indicated by lower externalizing problems and ADHD symptoms. Thus, our findings are consistent with empirical findings linking limited and unpredictable material support with lower directed exploration (Xu et al., 2023). Importantly, although directed exploration might incur costs in the form of limited procurement of immediate resources, it is essential for effective learning and the acquisition of advanced, complex skills that may reap larger benefits over longer time spans (e.g., Frankenhuis & Gopnik, 2023; Gopnik, 2020). Interpreted within a life-history framework, the privilege afforded to children in more resourced socialization contexts may be instantiated in slow-life strategies that encompass future-oriented resource acquisition tactics (i.e., direct exploration) and lower-risk behaviors.

Finally, findings for the balanced exploration/exploitation profile supported the conclusion that it offers a moderate (or “appropriate)” level of uncertainty-directed exploration that yielded the highest rewards among all profiles through the use of exploitative strategies. Also consistent with this conclusion, this profile exhibited the lowest random exploration. Children in this profile seemed to exhibit lower ADHD symptoms compared to the high-random exploration profile. Although significant differences comparing this profile versus others are limited in the current study, we speculate that this profile may potentially align with the hidden talent framework (i.e., showing advantages than others in learning where to locate the rewards; Frankenhuis et al., 2020; similar to findings in Li et al., 2023), and the accelerated exploration–exploitation shift hypotheses (i.e., reflecting an earlier shift to exploitation from heightened exploration; Frankenhuis & Gopnik, 2023). However, given the small size of this latent profile and the lack of significant findings, we encourage future research to further investigate its existence and implications within the exploration–exploitation framework.

While our findings across these profiles generally align with prior research (e.g., Hanson et al., 2017; Xu et al., 2023), synthesizing our findings with the broader literature requires careful consideration of the significant methodological heterogeneity in how exploration–exploitation is operationalized. Researchers have utilized a diverse array of paradigms, including the balloon emotional learning task (Humphreys et al., 2015), probabilistic learning task (e.g., Hanson et al., 2017), foraging task (e.g., Lenow et al., 2017; LIoyd et al., 2022), and the umbrella of multi-arm bandit task (e.g., horizon task: Somerville et al., 2017; Xu et al., 2023; reward probability task: Davies et al., 2022). These tasks place varying demands on cognitive resources. For example, foraging tasks require searching in infinite horizons within a certain time, whereas our reward probability task operates as a finite-choice bandit paradigm that requires balancing information gain against reward across a set number of discrete trials. Despite these differing cognitive demands, it is encouraging that our findings regarding socioeconomic risk and heightened random exploration converge with prior work utilizing a probabilistic learning task (e.g., Hanson et al., 2017).

Several limitations are worth mentioning. First, our sample consisted of families with elevated socioeconomic adversity compared to the general US population but potentially having more resources than our evolutionary past as a species. Thus, how much these findings generalize to children who face more economic extremes is not yet clear. Furthermore, although we consider our study in young children as a strength, the age-related constraints (e.g., attention span) limit our ability to implement more complex tasks and modeling approaches (e.g., Schulz et al., 2019). Relatedly, although our task and computational modeling strategies have been instantiated in prior literature for children at the same age (or even younger), previous works show that the computational parameters of young children might be more variable (e.g., Blanco & Sloutsky, 2024; Giron et al., 2023) and thus the interpretation of the present findings warrants consideration for the age group assessed. Because this age-related variability can include moments of task disengagement or difficulty that violate standard reinforcement learning assumptions, establishing upper parameter bounds (e.g., setting maximum values to 20) is a standard practice to ensure model stability. Second, although we adopted the longitudinal design, none of the key study components (e.g., reward probability task) were repeatedly measured. This correlational design limits our ability to illuminate the transition among different strategies (e.g., the shift from exploration to exploitation) described by Frankenhuis and Gopnik (2023). We encourage future studies to follow children over different developmental stages (e.g., early & middle childhood to adolescence) to illuminate the developmental trajectory of exploration–exploitation strategies. Third, future research would benefit from adopting multi-informant batteries in addition to our measure via teacher report for child functioning. Taken together, we adopted computational modeling and a person-centered approach to characterize child exploration–exploitation profiles. Family socioeconomic risk was associated with membership in the exploration–exploitation profiles, and these profiles were linked to differences in children’s socioemotional functioning. Findings highlighted exploration–exploitation patterns as potential processes through which children adapt to an adverse family context.

## Supporting information

10.1017/S0954579426101734.sm001 Li et al. supplementary material Li et al. supplementary material

## Data Availability

The raw data and study material are not publicly available due to ethical considerations for protecting participants’ privacy, but they are available upon request to the corresponding author. Analysis scripts: analysis scripts and output are publicly available in OSF (https://osf.io/pfxac/overview).

## Supplementary material

The supplementary material for this article can be found at http://doi.org/10.1017/S0954579426101734.

## Preregistration statement

The study is exploratory and thus not preregistered.

## Table 1. Long description

A matrix displays the bivariate correlations among eight primary study variables labeled 1 to 8: Uncertainty-Directed Exploration (beta), Random exploration (tau), Total Rewards Obtained, Public Assistance, Earned Income, Externalizing Symptoms, ADHD Symptoms, and Social Competence.

Navigate back to Table 1..

## Table 2. Long description

The table compares statistical fit indices across 1-Profile, 2-Profile, and 3-Profile Latent Profile Analyses model solutions for a sample size of 214 children based on their indices of directed exploration(beta), random exploration (tau), and total rewards obtained. A 4-Profile solution failed to converge. Fit metrics listed for the 1-, 2-, and 3-profile solutions include AIC, BIC, and ABIC, Entropy, and significance tests (LMR, VLMR, and B-LMR). The table also included information for profile percentage distribution for each solution.

Navigate back to Table 2..

## Table 3. Long description

Logistic regression results are evaluated across three latent classes (N = 214) using alternating baseline comparison classes. In the first block, Profile 3 (High Random Exploration) serves as the comparison class. In the second block, Profile 1(Balanced Exploration/Exploitation) serves as the comparison class. Greater received public assistance was linked to lower likelihood for children to be classified into Profile 2(High Directed Exploration) compared to Profile 3(High Random Exploration).

Navigate back to Table 3..

## Table 4. Long description

The table details child socioemotional functioning (N = 214) across three latent profiles: Profile 1 (Balanced Exploration/Exploitation), Profile 2 (High Directed Exploration), and Profile 3 (High Random Exploration), providing the Mean and Standard Error for each group alongside pairwise chi-square contrast tests results.

Navigate back to Table 4..
