# Supplementary material for: Family context, directed and random exploration profiles, and child socioemotional functioning
Source: Dev Psychopathol. 2026 Jul 17:1–12. Online ahead of print. doi: 10.1017/S0954579426101734 (PMC13423597; doi:10.1017/S0954579426101734)
Supplement: Li et al. supplementary material [file S0954579426101734sup001.docx]

**Supporting Information**

**Additional details about randomization for the reward probability task**

We provide some additional information for the pseudo randomization of the reward probability task, which maintains a fairly equitable distribution of rewards through different blocks of the task. First, within the whole task, reward did not occur in the same tree location for greater than equal to five consecutive trials. Second, within each block (i.e., 20 trials in total), the majority tree had a winning rate of 70%, and the other two trees each had a reward rate of 15%. To achieve this, each 20-trial block was divided into two sub-blocks (i.e., 10 trials per sub-block). Within each sub-block, the majority had reward for seven times, and the other two non-majority tree switched in winning time of 1 vs. 2 (e.g., subblock 1: 7/1/2; subblock 2: 7/2/1 across the three trees).

**Additional Rationales about Modeling specifics**

We followed the Bayesian Upper Confidence Bound (Bayesian-UCB) sampling strategies described in Schulz et al. (2019) and Meder et al (2020), with the following modification to accommodate the nature of the task. That is, given our multi-arm bandit task (i.e., three tree locations) did not involve the complexity of the grid-search task (i.e., 8*8 = 64 arm-bandit), we did not employ the Gaussian Process (GP) model that leverages a kernel function to represent the covariances in input spaced based on distances, thereby handling spatial correlations of reward in high-dimension search spaces. As such, we simplified the sampling processes to B-UCB values for the three tree locations based on the weighted sum function of posterior expected reward value and the uncertainty of the location, as described in the manuscript.

**Rationale for Latent-Profile Model Selection**

As shown in the manuscript Table 2, We explored a four-profile solution but found it to be empirically inadmissible and unidentified, even after fitting the model with a high number of random starts (i.e., 5,000 initial-stage starts) to ensure global optimization. Specifically, the four-profile solution failed to converge on a trustworthy solution, yielding a non-positive definite first-order derivative product matrix and a near-zero condition number (-0.457D-16). Furthermore, inspection of the parameters revealed that the model attempted to extract a spurious “extreme” profile representing only 3.27% of the sample (*N* = 7). Within this profile, the means for Beta hit the theoretical maximum of the scale (i.e., 20.00), resulting in undefined standard errors and thus Z-values. Given that the four-profile solution was statistically unidentified and mathematically unstable despite rigorous estimation attempts, we did not select the four-profile solution (Marked as “did not converge” in the manuscript, Table 2). Furthermore, we did not continue to explore profile solutions with more than four profiles, given that the smallest profile in the four-profile solution was already quite small (i.e., 3.27%).

We selected the three-profile solution because (a) it yielded the lowest AIC, BIC, and ABIC values; (b) the entropy value was high (i.e., >.80), suggesting sufficient classification accuracy; (c) Although the LMR and VLMR was marginally to non- significant, bootstrapped LMR test achieved significance, suggesting that the three-profile solution was superior to the two-profile solution; (d) the three-profile solution yielded three profiles that are more conceptually meaningful (i.e., high-directed exploration, high-random-exploration, balanced exploration/exploitation profiles).

Table S1. Demographic Characteristics of the Sample.

|  |  | | *n* | *%* |
| --- | --- | --- | --- | --- |
| **Child Sex** | Girls | | 136 | 56.0% |
| **Child Race** | Black or African American | | 111 | 45.7% |
|  | White | | 95 | 39.1% |
|  | Biracial (White and Black) | | 31 | 12.8% |
|  | Asian | | 2 | 0.8% |
|  | Other | | 4 | 1.7% |
| **Parent Marital Status** | Single/Never Married | | 25 | 10.3% |
|  | Married | | 119 | 49.0% |
|  | Divorced | | 2 | 0.8% |
|  | Separated | | 1 | 0.4% |
|  | Cohabiting | | 96 | 39.5% |
| **Maternal Highest Education** | Below 7^th^ Grade | | 1 | 0.4% |
|  | 8^th^-9^th^ Grade | | 7 | 2.9% |
|  | 10^th^-11^th^ Grade | | 32 | 13.2% |
|  | GED or High School Diploma | | 62 | 25.5% |
|  | Vocational Degree or Associates Degree | | 82 | 33.7% |
|  | Bachelor’s Degree | | 34 | 14.0% |
|  | Master’s Degree or Doctoral Degree | | 25 | 10.3% |
|  | *M* | *SD* | *Min* | *Max* |
| **Child Age @ Wave 3** | 6.77 | 0.48 | 5.83 | 8.33 |

Table S2. Latent Profile Solution for the Three-Profile Model.

|  | *Estimates(SE)* | *t* | *p* |
| --- | --- | --- | --- |
| Profile 1(Balanced Explore/Exploit) |  |  |  |
| Uncertainty-directed Exploration (*β*) | 3.74(0.48) | 7.73 | .00 |
| Random Exploration (*τ*) | 0.83(0.08) | 10.35 | .00 |
| Total Rewards Obtained | 48.15(1.00) | 48.16 | .00 |
| Profile 2(High Directed Exploration) |  |  |  |
| Uncertainty-directed Exploration (*β*) | 19.41(0.17) | 116.91 | .00 |
| Random Exploration (*τ*) | 3.14(0.22) | 14.39 | .00 |
| Total Rewards Obtained | 42.25(0.50) | 84.60 | .00 |
| Profile 3 (High Random Exploration) |  |  |  |
| Uncertainty-directed Exploration (*β*) | 4.49(1.60) | 2.81 | .005 |
| Random Exploration (*τ*) | 19.70(0.22) | 91.26 | .00 |
| Total Rewards Obtained | 36.85(1.02) | 36.12 | .00 |

Table S3. Logistic Regression Coefficients for Three-class Solution Using Adjusted Earned Income as Predictor (*N* = 212).

|  |  | *Coefficient (SE)* | *Z* | *p* |
| --- | --- | --- | --- | --- |
| Profile 3 (High Random Exploration) as Comparison Class | | | | |
| Profile 1(Balanced Exploration/Exploitation) | Earned income (Adjusted for Family Size) | -0.06(0.05) | -1.20 | .23 |
|  | Public Assistances | -0.05(0.03) | -1.77 | .08 |
| Profile 2(High Directed Exploration) | Earned income (Adjusted for Family Size) | -0.03(0.04) | -0.76 | .45 |
|  | Public Assistances | -0.07(0.03) | -2.70 | .01 |
| Profile 1(Balanced Exploration/Exploitation) as Comparison Class | | | | |
| Profile 2(High Directed Exploration) | Earned income (Adjusted for Family Size) | 0.02(0.03) | 0.70 | .48 |
|  | Public Assistances | -0.03(0.02) | -1.34 | .18 |

*Note.* This table presents analyses in Table 3 while replacing the total earned income with the total earned income adjusted for family size (i.e., adjusted earned income= total earned income/ [total # adults + children living in the household]).

Table S4. Logistic Regression Coefficients for Three-class Solution Including Additional Demographic Covariates (*N* = 212).

|  |  | *Coefficient (SE)* | *Z* | *p* |
| --- | --- | --- | --- | --- |
| Profile 3 (High Random Exploration) as Comparison Class | | | | |
| Profile 1(Balanced Exploration/Exploitation) | Earned income (Adjusted for Family Size) | -0.01(0.01) | -1.27 | .20 |
|  | Public Assistances | -0.04(0.03) | -1.52 | .13 |
|  | Child Age in Months at Wave 3 | 0.05(0.04) | 1.14 | .26 |
|  | Child Race | -0.25(0.22) | -1.12 | .26 |
| Profile 2(High Directed Exploration) | Earned income (Adjusted for Family Size) | -0.004(0.01) | -0.39 | .70 |
|  | Public Assistances | -0.07(0.03) | -2.41 | .02 |
|  | Child Age in Months at Wave 3 | -0.01(0.04) | -0.12 | .90 |
|  | Child Race | 0.22(0.21) | 1.05 | .30 |
| Profile 1(Balanced Exploration/Exploitation) as Comparison Class | | | | |
| Profile 2(High Directed Exploration) | Earned income (Adjusted for Family Size) | 0.01(0.01) | 1.23 | .22 |
|  | Public Assistances | -0.03(0.02) | -1.34 | .18 |
|  | Child Age in Months at Wave 3 | -0.06(0.03) | -1.82 | .07 |
|  | Child Race | 0.46(0.15) | 3.16 | .002 |

*Note.* This table presents findings of socioeconomic indicators in association with profile memberships while controlling for additional demographic covariates (i.e., child age in months at Wave 3 when child completed the reward probability task; and child race [see frequency distribution in Table S1]).


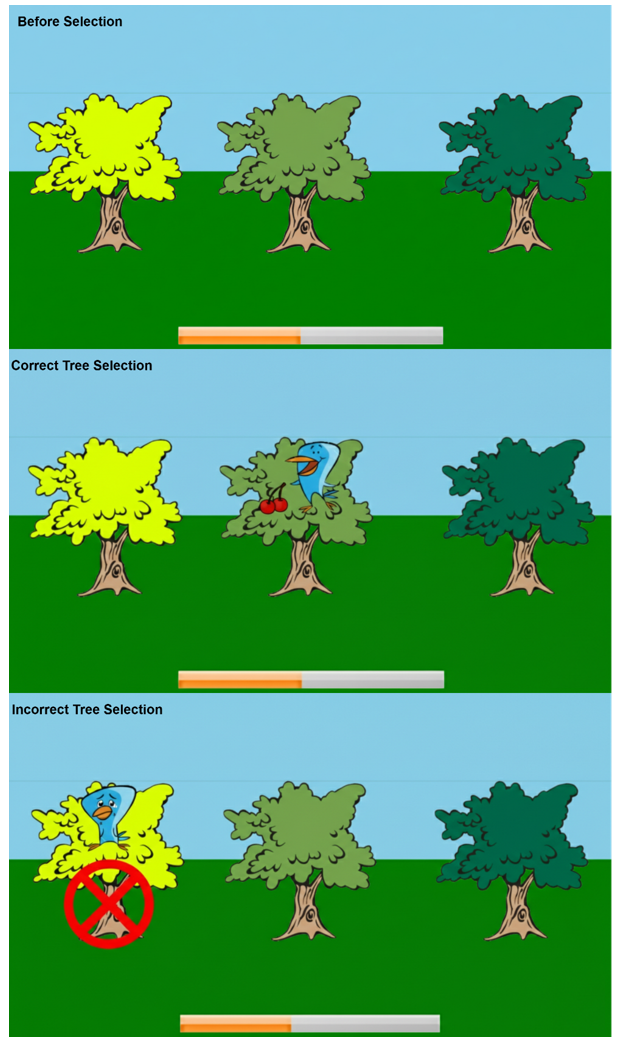


Figure S1. Illustration for the Reward Probability Task.

*Note*. The first picture depicts the three trees presented to the children; the second shows an example of a correct selection, while the third illustrates an incorrect selection.


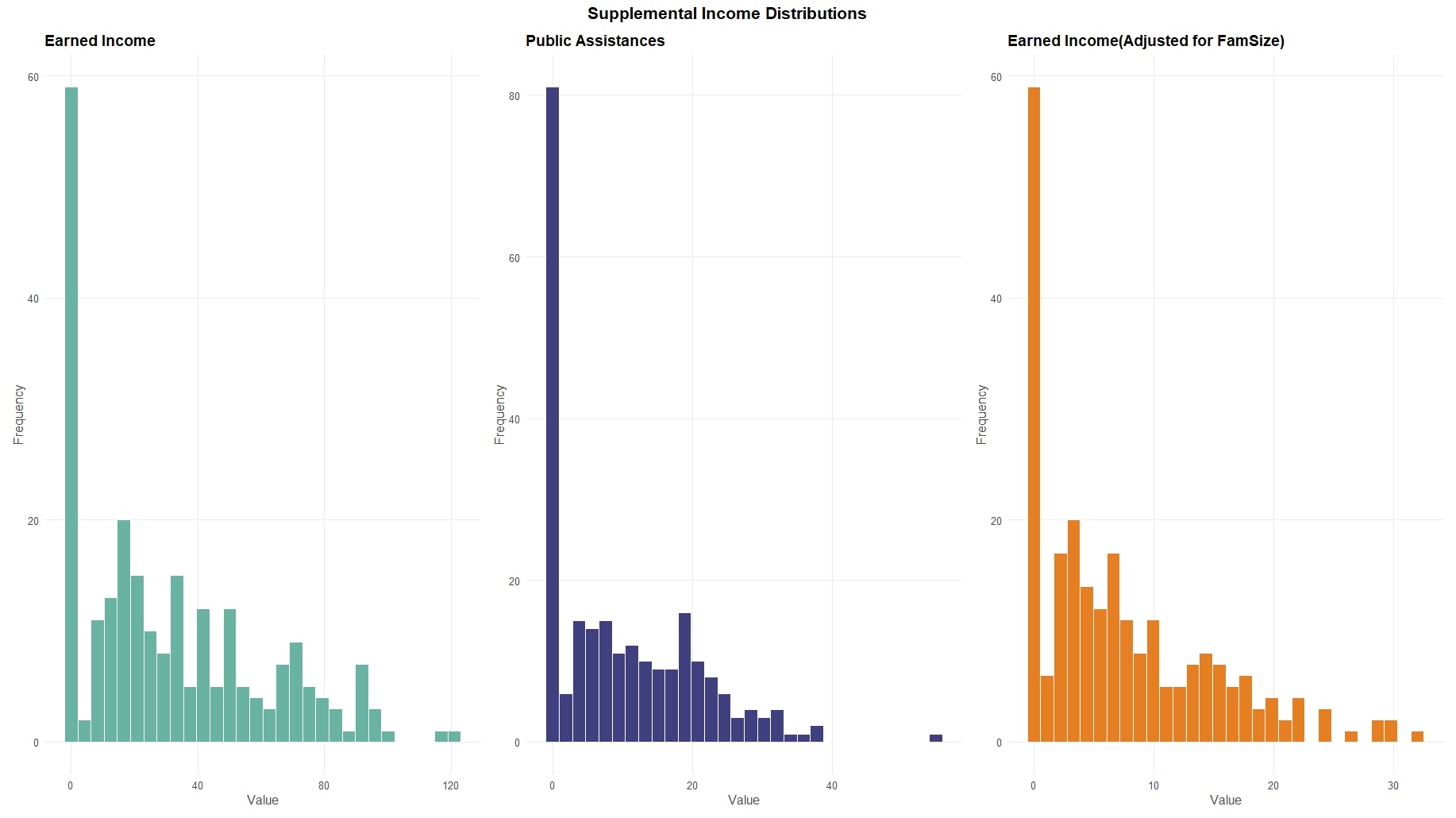


*Figure S2*. Histograms of the earned income, public assistance, and the earned income adjusted for family size.
